# Laser Chemosensor with Rapid Responsivity and Inherent Memory Based on a Polymer of Intrinsic Microporosity

**DOI:** 10.3390/s110302478

**Published:** 2011-02-28

**Authors:** Yue Wang, Neil B. McKeown, Kadhum J. Msayib, Graham A. Turnbull, Ifor D. W. Samuel

**Affiliations:** 1 Organic Semiconductor Centre, SUPA, School of Physics and Astronomy, University of St. Andrews, St. Andrews, Fife KY16 9SS, UK; E-Mail: yw244@st-and.ac.uk (Y.W.); 2 School of Chemistry, Cardiff University, Cardiff CF10 3AT, UK; E-Mails: mckeownnb@cardiff.ac.uk (N.B.M.); msayibk@cardiff.ac.uk (K.J.M.); 3 School of Physics & Astronomy, University of St Andrews, St Andrews, Fife KY16 9SS, UK

**Keywords:** polymer of intrinsic microporosity, organic semiconductor, distributed feedback laser, explosive detection, chemosensors

## Abstract

This work explores the use of a polymer of intrinsic microporosity (PIM-1) as the active layer within a laser sensor to detect nitroaromatic-based explosive vapors. We show successful detection of dinitrobenzene (DNB) by monitoring the real-time photoluminescence. We also show that PIM-1 has an inherent memory, so that it accumulates the analyte during exposure. In addition, the optical gain and refractive index of the polymer were studied by amplified spontaneous emission and variable-angle ellipsometry, respectively. A second-order distributed feedback PIM-1 laser sensor was fabricated and found to show an increase in laser threshold of 2.5 times and a reduction of the laser slope efficiency by 4.4 times after a 5-min exposure to the DNB vapor. For pumping at 2 times threshold, the lasing action was stopped within 30 s indicating that PIM-1 has a very fast responsivity and as such has a potential sensing ability for ultra-low-concentration explosives.

## 1. Introduction

There is currently a critical need for trace explosive detection due to the increased security issues and threats across the world. High-sensitivity detection techniques are needed to avoid missing potentially dangerous objects during routine checks at airports, government buildings and other places at risk of being targeted by terrorists. These devices could also be employed for detecting landmines. A range of optical spectrometric and chemical analytical methods have been investigated for this application [1]. Detection limits at the femtogram level, or a few parts per billion (ppb) by volume, of explosives such as trinitrotoluene (TNT) have been achieved using a number of different techniques. Research in this field continues to try to improve detection limits, speed of responsivity and portability.

Fluorescent polymers are one promising approach for trace explosive sensors [2–6], in particular for sensing nitro-aromatic based explosives. The highly nitrated molecules in these explosives make them electron-deficient so that when these electron acceptors are in contact with electron-rich semiconducting polymers, photoinduced electron transfer occurs between the lowest unoccupied molecular orbital (LUMO) of the polymer and the LUMO of the analyte molecule with lower energy state. This charge transfer causes efficient quenching of the fluorescence. The use of polymer thin films enables a quenching enhancement as a single explosive molecule can quench many chromophores, both on the same and neighboring polymer chains. However, a disadvantage of a solid film is that diffusion of the analyte into the film can be very slow.

In this paper we explore the effect of using a microporous polymer to address this issue—a soluble polymer of intrinsic microporosity (polybenzodioxane PIM-1) [7,8]. The microporosity of the polymer forms as a result of the rigidity of the macromolecular chain and its contorted structure [9]. This polymer and related PIMs have been used in the past as robust gas separation membranes [10–13], a trace organic volatiles indicator [14] and a pre-concentrator trap for trace explosive vapors [15]. For this investigation, we show that PIM-1 can be used as a light-emitting material within a sensor for trace vapours of nitro-aromatic molecules. The porosity in PIM-1, which aids the penetration of the analyte molecules, has potential to allow rapid responsivity and ultra-low concentration detection. We show that photoluminescence (PL) from the polymer is quenched by the introduction of the vapours and explore its ability to accumulate the analyte.

In addition, stimulated emission from the polymer allows enhancement of the detected signals, which is critical for successfully sensing trace amounts of explosives. As well as studying PL quenching, we also explore the feasibility of making a laser from PIM-1 and using it for explosive detection and investigate the change in laser output due to the analyte binding to the gain medium. This approach has been used successfully for solid films [3,4,16] but data on response time is extremely limited.

## 2. Experimental Section

**Figure 1.**
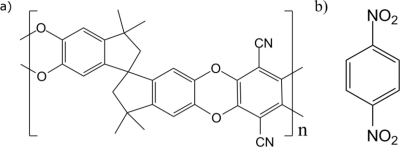
**(a)** Molecular structure of PIM-1; **(b)** Molecular structure of 1,4-dinitrobenzene (DNB).

The nitroaromatic analyte used in the investigation was 1,4-dinitrobenzene (DNB)—which we use as a model for the explosive TNT due to their similar characteristics (*i.e.*, nitro-aromaticity and strong electron-deficiency [2]). The chemical structures of PIM-1 and DNB are shown in Figure 1. A PIM-1 film of 340 nm thickness (measured with a Veeco Dektak 150 surface profiler) was spin-coated from chloroform solution (30 mg/mL) onto a planar fused silica substrate. Absorption and PL spectra were recorded using a Cary 300 Bio UV-Vis spectrophotometer and a HORIBA Jobin-Yvon FluoroMax2 fluorometer, respectively. The photoluminescence quantum yield (PLQY) in solid state was measured using an integrating sphere [17] in a Hamamatsu Photonics C9920-02 measurement system [18].

**Figure 2.**
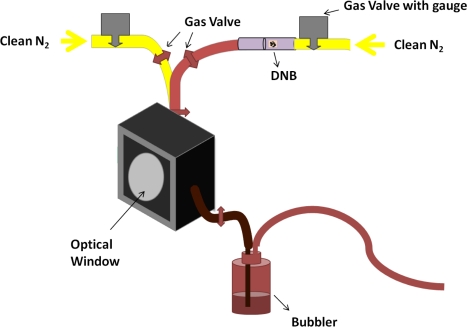
Sketch of the experimental set-up.

In the photoluminescence (PL) sensing experiments, the PIM-1 thin films were excited by a pulsed Nd:YVO_4_ microchip laser at 355 nm at 1 kHz repetition rate and the emission spectra from the films were monitored with a fibre-coupled grating spectrograph and a charge-coupled device (CCD) detector. To facilitate exposure to DNB, the PIM-1 thin films were placed inside an optical chamber connected to a gas port with two gas supplies allowing us to flow either DNB vapor or clean nitrogen over the sample, as shown in Figure 2. An exhaust port from the chamber was connected to a bubbler. This configuration has been shown to give a DNB vapor pressure of ∼10 ppb by volume [4].

To measure the dynamic response of the sensing process, the chamber was first purged with clean nitrogen and the gas port was then switched to the DNB vapor. For continuous exposure experiments the dynamics of the PL emission were monitored for the first 3 min. The emission from the same film was then also measured after a 30-min exposure. Additionally, a cumulative PL sensing scheme was achieved by monitoring the PL emission while switching the gas flow between clean nitrogen and DNB vapor repetitively—alternating the chamber atmosphere between low (∼0 ppb) and ‘high’ (∼10 ppb) DNB.

In order to assess the potential of PIM-1 as a laser gain medium, we measured the amplified spontaneous emission (ASE) from the edge of the films using the fibre-coupled CCD spectrograph. Optical excitation was from a Nd:YAG laser pumped optical parametric oscillator (OPO) at 425 nm with 4 ns output pulses with a repetition rate of 20 Hz. A series of calibrated neutral density filters were inserted into the beam path to vary the excitation pulse energy. A cylindrical lens was used to focus the pump beam into a 170 μm wide stripe with a length of 3.19 mm (measured with a Coherent Inc. Beam Profiler) on the surface of the polymer films.

The refractive index of PIM-1 films was measured using variable angle spectroscopic ellipsometry (a J. A. Woollam Co. Inc. M2000-DI ellipsometer was used). A set of seven films of different thickness in the range of 75 nm to 192 nm were prepared by spin coating a PIM-1 solution (chloroform, 20 mg/mL) onto fused silica substrates. The polarization of reflected light was measured for incident angles between 45° and 75° in steps of 5° over the wavelength range of 190 nm to 1,700 nm. Transmission measurements at normal incidence were also taken. Both isotropic and uniaxial Cauchy models were then fitted to the combined reflection and transmission data between 500 nm to 1,700 nm to determine the thickness of the films. With these known thicknesses the refractive index was calculated across the wavelength range of interest. The effective refractive index of the air-polymer-SiO_2_ waveguide was also calculated to determine the optimal distributed feedback (DFB) laser configuration.

In order to fabricate a surface emitting DFB laser, a thin film was deposited by spin coating from a 30 mg/mL PIM-1 solution in chloroform onto a corrugated fused silica substrate. These corrugations, formed by two-beam holography provide laser feedback (via second order diffraction) and surface output coupling (via first order diffraction) at a specified wavelength, determined by the Bragg condition [19]:

(1) $${m\lambda }_{\mathrm{Bragg}}={2n}_{\mathrm{eff}}\Lambda \;\;\;\;(m=2)$$

*λ_Bragg_* being the wavelength of the light, *n_eff_* the effective refractive index of the thin film waveguide and *Λ* the period of the corrugated grating. The period of the one-dimensional second-order DFB resonators in the fused silica substrate was 350 nm and the grooves were made 50 nm deep with fill factor of approximately 50%. The PIM-1 laser was optically pumped with 425 nm pulses at 20 Hz repetition rate from the Nd:YAG laser pumped OPO. The pump beam was incident onto the samples at an angle of 20° to the surface normal and was focused to a 1.5 mm diameter spot on the polymer laser; this resulted in an excitation area of 0.0177 cm^2^. The fibre-coupled CCD spectrograph was used to monitor the dynamics of the laser emission perpendicular to the PIM-1 sample in steps of 10 s. In addition, the laser thresholds and slope efficiencies were measured, both before exposure and after a 5-min exposure to 10 ppb DNB vapor.

## 3. Results and Discussion

### 3.1. Sensing of Explosives Based on Photoluminescence of PIM-1

**Figure 3.**
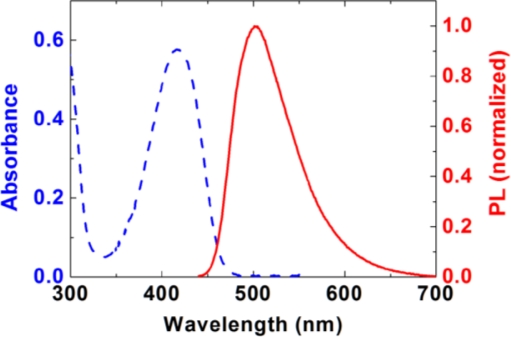
Absorption (dashed line) and PL spectra (solid line) of a 340 nm thick PIM film.

Figure 3 shows the absorption and PL (excited at 400 nm) spectra from a 340 nm thick PIM-1 film. The absorption has a maximum at 416 nm and the emission spectrum is peaked at 501 nm. The PLQY of an as-spin-cast PIM-1 film was measured to be 21%.

**Figure 4.**
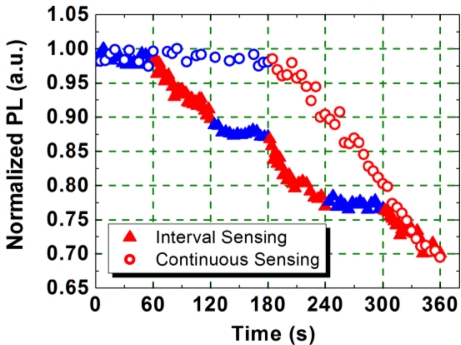
Both interval (triangles) and continuous (open circles) PL sensing with a 340 nm thick PIM film (red highlights sensing, blue highlights clean nitrogen purge).

When the 340 nm thick PIM-1 film, excited at 355 nm, was exposed to the DNB vapor, the PL emission was quenched. The dynamics of the PL quenching are shown in Figure 4 (open circles). Prior to DNB exposure, during the first 3-min nitrogen purge, the emission from the film is seen to be stable, but as soon as the film was exposed to the DNB vapor the emission reduced. After a 3-min exposure, the emission intensity was reduced by 31%, and after a 5-min exposure, the emission intensity was reduced by a further 8%. After 30-min continuous exposure, the emission intensity was reduced by 85%, this being the sensing efficiency, defined as the change of the emission intensity during exposure over the initial intensity. A similar change in the PLQY of the PIM-1 film was also observed. It decreased from 21% (pre-exposure) to 3% (post-exposure).

In the cumulative sensing test, the sample was first exposed to nitrogen vapor for 60 s, and then exposed to DNB vapor for 60 s leading to a PL intensity drop of 10% (shown in Figure 4). In the following 60-s nitrogen purge the emission remained unchanged. Further quenching of the PL emission resumed when the film was exposed to a second flow of DNB vapor. After a total of 3-min exposure the PL intensity was reduced to the same level as the continuous 3-min exposure. This behavior is different from other fluorescent polymer sensors, where the light emission recovers to its original level when the polymer film is isolated from the source of the nitroaromatic vapour. This suggests that the interaction between the electron-rich polymer PIM-1 and the electron-deficient DNB molecules is significantly stronger than for other light-emitting polymers. This inability to recover has been confirmed by using a vacuum pump to evacuate the sensing chamber (vacuum of 10^−4^ mbar). The intensity of emission was still found not to increase during a 15-h evacuation (data not shown). This indicates a strong interaction between the electron-rich polymer and the electron-poor analyte. The accumulation of the DNB in the PIM-1 film offers the potential to sense very low concentrations of vapours over time and allows an inherent memory of exposure history for the polymer PIM-1, which may be useful for application in shipping and storing of sensitive items or in the measurement of total exposure time. Our studies were all performed at room temperature, but it has been shown that the binding of explosive vapor to PIM-1 can be reversed by heating [15].

### 3.2. Study of Gain and Optical Constants of PIM-1

**Figure 5.**
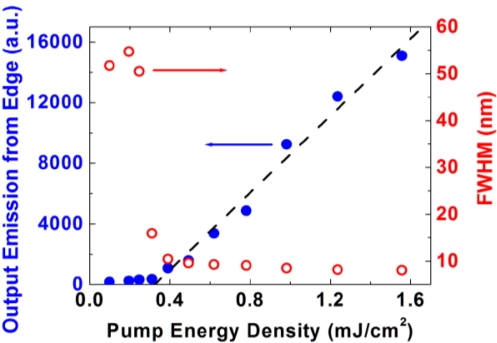
The edge emission intensity from a PIM-1 planar waveguide as well as the dependence of the FWHM of the emission spectra *versus* pump energy density.

We next assessed the potential of PIM-1 for generating laser light. A 340 nm thick PIM-1 film was optically excited with the pulsed output from the OPO focused to a narrow stripe and the emission from the edge was monitored using the CCD spectrograph. We observed the characteristic spectral narrowing of the light emission due to amplified spontaneous emission (ASE), with a peak wavelength at 528 nm. Figure 5 shows the full-width at half-maximum (FWHM) linewidth of the emission spectrum and relative output intensity, as a function of the pump intensity. We found that the FWHM dropped dramatically from 52 nm to 8 nm when pumping above a threshold intensity of 310 μJ/cm^2^. The reduction in linewidth occurs due to the net gain being maximal near the peak of the PL spectrum and thus the spectrum exhibits gain narrowing as the pump intensity increases.

The experimental data from variable angle spectroscopic ellipsometry were fitted with both isotropic and uniaxial Cauchy models. The isotropic model fitting yielded an excellent agreement between the experimental data and the model generated data, indicating that the PIM-1 molecules are arranged in an unordered homogeneous fashion within the film. The uniaxial fit to the data gave a high agreement to the ordinary and extraordinary refractive indices, confirming that the polymer films are isotropic. The refractive index *n* of PIM-1 at 528 nm (the peak of ASE), was found to be 1.59 from the optical constants fitting. This value is lower than conventional semiconducting conjugated polymers [20,21], which reflects the relatively low density of the PIM-1 films due to its microporous structure. The effective refractive index of the guided TE_0_ mode was calculated for the air-polymer-silica waveguide structure [22] and found to be 1.49.

### 3.3. Sensing of Explosives Based on PIM-1 DFB Lasers

We next fabricated surface-emitting PIM-1 DFB lasers on a 1D 350 nm period grating. Knowing the effective refractive index of the waveguide we were able to design the DFB laser to operate at an emission wavelength of 529 nm (overlapping with the peak of the ASE spectrum) by controlling the thickness of the film. The laser had a threshold pump density of 174 μJ/cm^2^, above which the laser emission increased linearly with pump density.

**Figure 6.**
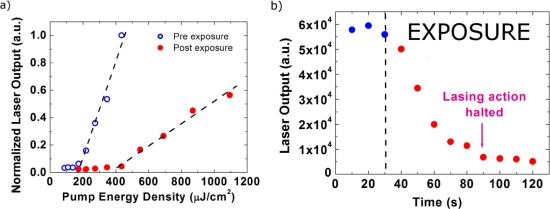
**(a)** Power characteristics of a PIM laser before and after a 5-min exposure; **(b)** Laser output dynamics during a 90-s exposure.

The laser was then exposed to ∼10 ppb DNB vapor. Figure 6(a) shows the laser characteristics both before and after a 5-min exposure. After the exposure, we found the laser threshold increased to 436 μJ/cm^2^, which is 2.5 times higher than the pre-exposure threshold. A reduction of 4.4 times in the slope efficiency was also observed. When setting the excitation energy close to the threshold of the exposed laser a maximum sensing efficiency (as defined above) of 95% was achieved, which is 2.4 times higher than the PL sensing efficiency under the same exposure conditions.

Figure 6(b) shows the dynamics of the laser emission during exposure to the DNB vapor with the excitation energy set to twice the threshold of the pre-exposed laser, *i.e.*, 346 μJ/cm^2^. We observed that the laser output decreased rapidly (60% within 30 s). Laser action was seen to halt after a 60 s exposure, which is why the emission decay slows down at this point, the sensing efficiency decreases significantly when only spontaneous emission can be measured. The rapid responsivity and high sensitivity of the PIM-1 may be due to its open microporous structure and increased effective available surface area, which helps penetration of the DNB molecules into the film. The pore size distribution of PIM-1 is mostly sub-nanometer with a maximum at 0.65 nm as determined using the Horvath-Kawazoe analysis of low pressure nitrogen adsorption at 77 K [11,13]. As shown in the Table 1, the PIM-1 laser sensor has higher sensing efficiency and much faster responsivity compared to the polyfluorene (PFO) sensor, previously reported [4].

## 4. Conclusions

We have demonstrated that the polymer PIM-1 is a good chemosensor for detecting low vapor pressure (∼10 ppb) DNB vapor. Successful indication of the presence of the analyte, by monitoring the photoluminescence has been achieved. The response accumulates suggesting a very strong interaction between DNB and the microporous polymer and leads to an inherent memory ability, which has the potential for applications requiring an integrated measurement of vapour concentration. These include the tracing of history of exposure during the shipping and storing of sensitive items, an exposure timer and an efficient explosives absorber [15]. Our results also show that the very convenient DFB laser geometry has a significantly enhanced sensing efficiency and much faster responsivity to low vapor pressure explosives detection. A maximum sensing efficiency of 95% was achieved. In conclusion, the combination of ease of processing into thin coatings, its intense fluorescence and microporous structure makes PIM-1 a promising material for use in the inexpensive rapid sensing of ultra-low-concentrations of explosives based on nitrated aromatic compounds.

## Figures and Tables

**Table 1.** Comparison between explosive sensing properties of PIM-1 and PFO laser sensors—both exposed to ∼10 ppb DNB for 5 min.

**Sensing Characteristics**	**PFO**	**PIM-1**
Increase in laser threshold	1.8×	2.5×
Decrease in slope efficiency	3×	4.4×
Maximum sensing efficiency	82%	95%
Reduction in laser emission (relative speed of responsivity)	40%/46 s	60%/30 s
